# Self-reported disrespect and abuse by nurses and midwives during childbirth in Tanzania: a cross-sectional study

**DOI:** 10.1186/s12884-020-03256-5

**Published:** 2020-10-06

**Authors:** Kana Shimoda, Sebalda Leshabari, Shigeko Horiuchi

**Affiliations:** 1 Graduate School of Nursing Science, St. Luke’s International University, 10-1 Akashi-cho, Chuo-ku, Tokyo, 104-0044 Japan; 2 School of Nursing, Muhimbili University of Health and Allied Sciences, P.O. Box 65004, Dar es Salaam, Tanzania

**Keywords:** Disrespect and abuse, Mistreatment, Maltreatment, Respectful maternity care, Quality care, Childbirth, Intrapartum care, Tanzania

## Abstract

**Background:**

Facility-based childbirth has increased globally. Unfortunately, there have also been reports of women experiencing disrespect and abuse by healthcare providers during childbirth. This study aimed to measure the prevalence of self-reported disrespect and abuse (D&A) by healthcare providers of women during childbirth in health facilities in Tanzania, and to clarify the factors related to D&A.

**Methods:**

A cross-sectional survey was conducted in public health facilities of three regions in Tanzania from September 2016 to October 2016. Nurses and midwives who had ever conducted deliveries completed a 22-item section about D&A and three sections about working conditions and environment. A model for predicting D&A based on several factors such as their characteristics, working conditions, and working environment was developed by conducting multiple regression analysis.

**Results:**

Thirty public health facilities in three regions within Tanzania were selected to reflect different levels of hospitals. Among 456 participants (nurses, midwives, and nursing assistants), 439 were included in the analysis. Average number of self-reported D&A out of 22 items was five, and nearly all participants (96.1%) reported enacting one form of D&A at the least and two forms of D&A at the most. About 25–44% of D&A items were in the forms related to women’s experiences with childbirth psychologically. Moreover, at least 10–30% of the participants enacted some form of D&A which could directly affect the well-being of mothers and babies. D&A scores increased with an increase in ‘working hours per week’ and ‘taking a break during evening shifts’. D&A scores decreased with an increase in the scores of the ‘two components of the Index of Working Satisfaction (professional status and interaction between nurses)’, and ‘any type of supervision for new nurse-midwives’.

**Conclusion:**

Most studies about D&A of healthcare providers previously focused on the reports of women. To our knowledge, this is the first report that focused on D&A reported by healthcare providers. Working conditions and systems including personal relationships with colleagues were both positively and negatively related to D&A of healthcare providers rather than the provider’s individual and facility structural characteristics.

## Background

On September 25, 2015, 17 new sustainable development goals (SDGs) were adopted by the United Nations’ member states as a 15-year global guide [1]. The new SDGs focus more strongly on equity and people-centeredness from a human rights perspective [2]. As the SDGs evolved from the Millennium Development Goals, improving maternal and perinatal mortality has remained as one of the unfinished agenda items.

To reduce maternal and perinatal deaths, a key strategy has been the utilization of health facilities with maternity care provided by skilled birth attendants. Thus, facility-based birth rates have increased particularly in low- and middle-income countries over the last two decades [1]. Although national strategies to improve maternal and newborn health have been given much attention particularly in areas such as health coverage or quantity of resources, the quality dimension has received less attention over the last decade [3]. In this context, the quality of facility-based care during childbirth has become an important subject of discussion among maternal and child health policy makers [4, 5]. Researchers have found that increasing institutional birth rates alone is insufficient in reducing maternal and perinatal mortality and morbidity. High-quality maternity care that supports robust health systems would be crucial to save the lives of women and newborns [6, 7]. In addition, improving the quality of care may not only provide physical safety care but also achieve a good childbirth experience that is memorable for years after birth [8, 9]. These good care and childbirth experiences may encompass respect for women’s basic human rights [10–12].

Unfortunately, mounting evidence exists indicating that women may experience disrespect and abuse (D&A) by healthcare providers during facility-based childbirth. Bowser and Hill’s landmark review of research about facility-based D&A found physical abuse, non-consented care, non-confidential care, non-dignified care, discrimination, abandonment and detention in facilities [13]. In Tanzania, similar negative treatment and women’s experiences have been reported during childbirth [14–17].

The prevalence of any D&A experiences reported by postpartum women was 12 to 70% [14, 17–19]. The most commonly reported D&A experiences were non-dignified care (i.e., shouting, scolding and threatening) and abandonment of care (i.e., being ignored and birth without attendants). Direct observation studies have indicated that almost all women (80–100%) did not provide consent for examinations, 5% experienced undignified language by providers and 6–60% were shouted at during history taking [17, 19].

A healthcare provider’s negative behavior is influenced by stress, fatigue, frustration and poor job satisfaction. These conditions are affected by facility level factors such as work conditions and environment as well as work-related factors such as heavy workloads, long working hours, weak supervision, poor relationships with co-workers and insufficient salary [10, 13, 20, 21].

Women’s underutilization of health facilities for childbirth has been reported to be related to D&A in these facilities [16, 22–25]. A key cause of this underutilization was the loss of trust between women and healthcare providers owing to poor treatment including disrespectful, abusive and neglectful care. These violations of trust between women and their healthcare providers create a disincentive for women to seek skilled attendance [18, 23]. Thus, there has been a growing concern among healthcare policy makers and clinicians regarding the quality of care during childbirth in health facilities in low- and middle-income countries.

WHO released a statement on “The prevention and elimination of disrespect and abuse during facility-based childbirth” in 2014. The statement indicated that there was still no internationally agreed definition or measurement tool of D&A [26].

To date, there has been little study concerning D&A and factors related to D&A. Most international qualitative and quantitative studies about D&A focused on women’s reports. There have been limited studies on D&A focused on providers’ perspectives. In this context, the present study aimed to measure the prevalence of D&A by nurses and midwives of women during childbirth in health facilities in Tanzania, and to determine factors related to D&A.

## Methods

### Study design, settings and participants

The study design was a descriptive retrospective cross-sectional survey using a self-completed questionnaire from nurses and midwives.

This study was conducted in three regions in Tanzania and included both urban and rural areas which have public referral hospitals that accepted the conduct of this research. At the time of this study, there were only four public referral hospitals in Tanzania. The three regions were Dar es Salaam region located in the Eastern zone, Mbeya region located in the South West Highlands zone and Mwanza region located in the Lake zone, with perinatal mortality rates per zone of 52 per 1000 live births, 38 and 36. Some important background information of these three regions are as follows: regional populations 5,156,000 (Dar es Salaam region), 3,728,000 (Mbeya region) and 4,131,000 (Mwanza region); numbers of births per year 772,000, 559,000 and 737,000; 94.2, 64.9 and 53.3% facility-based births [27]. All referral (*n* = 3) and regional (*n* = 5) hospitals and a convenient selection of district hospitals (*n* = 7) and health centers (*n* = 15) were selected from those regions as some of them did not conduct deliveries at that time. Only public hospitals where births took place were included because private and public health systems are different.

Nurses, midwives or nursing assistants who had ever conducted deliveries within the last 3 years, were eligible to participate. The research assistants (RAs) stayed in the participating hospitals almost every day from the morning to the evening shift. They individually explained to the nurses, midwives or nursing assistants the contents of the study after being introduced by the nursing officer-in-charge and then invited them to participate.

### Sample size

The sample size was estimated based on having five subjects for each item studied [28]. The Index of Working Satisfaction (IWS) scale by Stamp had 44 items, thus among other factors which are assumed to be related to D&A, the minimum sample size was estimated as 220 [29]. Considering a dropout rate of 30 and 15% missing values, sample size was set at 329.

### Measurements of job satisfaction and D&A

The questionnaire had four sections: sociodemographic characteristics, individual working conditions (commute, working hours and break, work shifts, overtime work, side jobs, salary, and busyness), the IWS of Stamp, and D&A behavior.

The IWS of Stamp was used to measure working satisfaction as negative behaviors of healthcare providers were influenced by stress, fatigue, frustration and poor job satisfaction. These conditions were affected by heavy workloads, insufficient salaries and poor relationships with co-workers [10]. The IWS is composed of 44 items that captures information about six components of job satisfaction as follows: (1) pay (dollar remuneration or fringe benefits), (2) autonomy (job-related independence, initiative and freedom, either permitted or required in daily work), (3) task requirements (as regular part of the job), (4) organizational policies (management policies and procedures from the hospital administration), (5) professional status (feeling about one’s job as well as in the view of others), and (6) interaction (opportunities for formal and informal social and professional contact during working hours) with two subscales: nurse-nurse interaction and nurse-physician interaction. Response choices of the 44 items used a seven-point Likert scale from 1 (*strongly disagree*) to 7 (*strongly agree*). Scores range from 44 to 308 points, and higher scores mean higher levels of job satisfaction. Cronbach’s alpha coefficients were calculated using five multiple imputation data sets (0.816, 0.818, 0.818, 0.818, and 0.819) with an average of 0.818 for overall scales that established internal consistency. The six subcomponent average coefficients ranged from 0.40 to 0.63.

The researcher developed all items for identifying D&A behavior of nurses and midwives. This was drawn from a literature review, including two key systematic reviews regarding D&A behavior during childbirth [13, 15, 17, 18, 20, 30–34]. Contents exemplifying D&A were extracted based on seven categories: 1) physical abuse, 2) non-consented care, 3) non-confidential care, 4) non-dignified care, 5) discrimination based on specific patient attributes, 6) abandonment of care, and 7) detention in facilities [13]. Category 8), unethical clinical practice, was derived and added from a previous study [33]. There are one to three empirical indicators or items for each of the eight categories yielding a total of 22 items, measured on a five-point Likert scale from 1 (*never)* to 5 (*always)*. All 22 D&A items were summed as total scores of those behaviors (ranged from 22 to 110). Higher scores indicated committing more D&A behaviors. Cronbach’s alpha coefficients were calculated using the five multiple imputation data sets. Cronbach’s alphas for these five datasets were 0.649, 0.657, 0.656, 0.656, and 0.653, and the average was 0.650, indicating moderate internal consistency.

All question items in English were translated to Kiswahili language in the questionnaire before collecting data. Two native Tanzanians performed the translation work from English into Kiswahili in four steps: 1) *translation* from English to Kiswahili; 2) *cross-checking* the translated questionnaire by comparing it with the original English version; 3) *back-translation* of the cross-checked questionnaire from Kiswahili into English to confirm the contents; and 4) *discussion* of the questionnaire contents with the two Tanzanians after the researcher compared the back-translated version with the original questionnaire. Of the two Tanzanians, one back-translated the questionnaire and the other participated in the second step of correcting and confirming the results. To establish face validity of the questionnaire, five midwives who had clinical experience working in health facilities responded to the questionnaire, and from their answers, the items were modified.

Additionally, a facility checklist for collecting data of the participating facilities was used to assess the health facilities where participating nurses and midwives have worked in terms of their working environment.

### Data collection and analysis

Data were collected between September 2016 and October 2016. Participants were informed about the purpose, methods and ethical considerations. The questionnaire and a self-seal opaque envelope were provided to the participants. After completing the questionnaire, sealed envelopes with the questionnaire could be returned in the nurse stations of their wards. Returning the completed questionnaire was regarded as agreement of participation. The time required for completing the questionnaire was 20 to 30 min. The RAs collected the envelopes during their shifts or in the next morning. Completed questionnaires were only opened by the researchers. Nursing officers in charge or ward gatekeepers provided data regarding the characteristics of their facility and obstetric statistics.

Data entry and analyses were performed using SPSS version 25 J statistical software. The null hypothesis, namely, data were ‘missing completely at random’ (MCAR), was rejected using Little’s MCAR test (*p* < 0.001). As the data were not MCAR, multiple imputation was performed [35]. As a result, 17 cases (one with > 50% missing data for all variables; 16 with already > 80% missing data for D&A) were excluded and two cases were pairwise deleted from the analysis because of missing data.

Descriptive statistics were used to summarize the background of the participants and facilities. Data of D&A behaviors were summarized using numbers (%) and frequency tables. To examine bivariate relationships between D&A behaviors and IWS, nurses’ and midwives’ working conditions and facility characteristics, *t*-test, one-way analysis of variance and calculation of Pearson’s correlation coefficient were used. To develop a model for predicting D&A of nurses and midwives based on several factors such as their characteristics, working conditions, and working environment, multiple regression analysis was used. Factors which were slightly correlated (*r* ≈ 0.2) with D&A behaviors’ total scores, associated with an increase or decrease in the score, and consistent with the conceptual framework of previous studies were used by forward selection after controlling simultaneously for potential confounders. The level of significance was two-sided at *p* < 0.05.

## Results

In this study, 456 questionnaires were distributed to nurses, midwives and nursing assistants who were eligible with a 100% response rate. Of the 439 total participants, 113 came from referral level hospitals, 116 from regional hospitals, 106 from district hospitals and 104 from health centers. As there was no large difference between the multiple imputation dataset and the original dataset, we only present the results after performing multiple imputation.

### Characteristics of participants and study settings

Mean age of the participants was 34.3 years with 8.7 years of experience as nurses and midwives. The majority (*n* = 390; 88.8%) had completed college (Table 1). Mean working hours per week was 49.4, and more than 60% worked regularly during night shifts. Regarding overtime work, only 7 over 33 (21%) in health centers were paid for overtime work compared with 34 over 53 (64.2%) in referral level hospitals and 32 over 55 (58.2%) in regional hospitals.

**Table 1:** Sociodemographic characteristics and participants’ working conditions (*N* = 439)

Variable	Response	Measure
Age in years, Mean (SD)		34.3 (±8.4)
Sex, n (%)	*Male*	54 (12.3)
	*Female*	385 (87.7)
Living with family members, n (%)	*Yes*	375 (85.4)
	*No*	64 (14.6)
Having to care for other persons in household, n (%)	*Yes*	363 (82.7)
	*No*	76 (17.3)
Having a house keeper at home, n (%)	*Yes*	287 (65.4)
	*No*	152 (34.6)
Level of educational attainment, n (%)	*College graduates or lower*	390 (88.8)
	*University graduates or higher*	49 (11.2)
Job status level	*Head / In-charge nurse*	129 (29.4)
	*Staff nurse / assistant*	310 (70.6)
Length of nursing experience in years, Mean (SD)		8.7 (±8.0)
Commute time to work *(minutes),* Mean (SD)		77.8 (±51.2)
Working hours per week *(hours),* Mean (SD)		49.4 (±22.4)
Working in night shift, n (%)	*Yes*	299 (68.1)
	*No*	140 (31.9)
Working overtime, n (%)	*Yes*	192 (43.7)
	*No*	247 (56.3)
Number of births conducted in the last month, Mean (SD)		25.0 (±27.9)
Number of days off per month *(days), n (%)*	less than 8 days	134 (30.5)
	8 days or more	305 (69.5)
Taking a break during evening shift	*Morning shift* *(6 h: 7・8 am - 1・2 pm), Mean (SD)* *(Range: 1=never - 5=always)*	1.7 (0.7)
	*Evening shift* *(6 h: 1・2 pm - 7・8 pm), Mean (SD)* *(Range: 1=never - 5=always)*	1.8 (0.7)
	*Night shift* *(12 h: 7・8 pm - 7・8 am), Mean (SD)* *(Range: 1=never - 5=always)*	1.1 (1.0)
Monthly salary *(USD)*, Mean (SD)		238 (±110.6)
IWS^a^ total score *(Range: 44 - 308)*		179.8 (26.2)

^a^*IWS* Index of Working Satisfaction

The regional level hospitals had the largest number of annual vaginal births (mean = 10,014 ± 4040) whereas the referral level hospitals had the highest number of annual cesarean sections (mean = 3114 ± 1943). The numbers of staff and beds were relative to the scale of each facility. There were few antenatal wards with partitions between beds (*n* = 8; 26.7%), although almost 19 (63.3%) of the facilities had partitions in the labor wards. The majority (*n* = 26; 86.7%) of the facilities had a rule restricting the right of women to have a birth companion during childbirth. Regarding in-service education, about 22 facilities (73.3%) had a supervision system for educating new nurses and midwives.

### Reported D&A behaviors

Table 2 shows D&A behaviors which were reported after performing multiple imputation. The majority of the participants (*n* = 422; 96.1%) had engaged in one of the 22 forms of D&A behavior at the least, and in two of the 22 forms of D&A behavior at the most: 5.20 ± 3.42 (range 0–20). Mean total score of D&A was 32.5 ± 7.134 (range 22–110). The most common D&A was “not draping women’s legs when performing vaginal examination” (*n = 290*; 66.0%).

**Table 2:** Frequency distribution of self-reported D&A behaviors toward women after performing multiple imputation (*N* = 439)

	Enacted					Not enacted
	Total ***N*** (%)	Always *n* (%)	Often *n* (%)	Sometimes *n* (%)	Seldom *n* (%)	Never *N* (%)
**Not draping women's legs when preforming vaginal examination**	**290 (66.0)**	140 (31.9)	67 (15.4)	42 (9.6)	40 (9.1)	149 (34.0)
**Not obtaining consent for performing episiotomy**	**203 (46.2)**	16 (3.6)	44 (10.0)	58 (13.1)	85 (19.4)	236 (53.8)
**Conducting deliveries even when many staff or students are present**	**189 (43.1)**	37 (8.5)	37 (8.3)	32 (7.3)	83 (19.0)	250 (56.9)
**Not telling the results of blood pressure reading**	**169 (38.6)**	4 (0.9)	29 (6.6)	39 (8.9)	97 (22.2)	270 (61.4)
**Blaming adolescent girls for being too young to get pregnant**	**164 (37.3)**	14 (3.2)	17 (3.8)	47 (10.8)	86 (19.5)	275 (62.7)
**Not using anesthesia for episiotomy or suturing the perineal tears**	**155 (35.4)**	29 (6.6)	26 (5.9)	19 (4.4)	81 (18.5)	284 (64.6)
**Scolding when women do not comply**	**122 (27.8)**	7 (1.6)	6 (1.4)	38 (8.7)	71 (16.1)	317 (72.2)
**Threatening when women do not comply**	**121 (27.6)**	13 (3.0)	19 (4.4)	28 (6.4)	61 (13.8)	318 (72.4)
**Not offering words of sympathy for women who suffer from labor pains**	**118 (27.0)**	7 (1.6)	6 (1.4)	10 (2.3)	95 (21.7)	321 (73.0)
**Preventing discharge until women completed payment**	**108 (24.5)**	14 (3.2)	21 (4.7)	29 (6.6)	44 (10.1)	331 (75.5)
**Asking women about their private information in public**	**104 (23.7)**	58 (13.2)	22 (5.0)	6 (1.4)	18 (4.1)	335 (76.3)
**Not obtaining consent for performing vaginal examination**	**102 (23.2)**	2 (0.5)	9 (2.1)	17 (3.9)	74 (16.8)	337 (76.8)
**Slapping women's legs to open during second stage of labor**	**92 (21.0)**	5 (1.1)	4 (0.9)	31 (7.1)	52 (11.9)	347 (79.0)
**Prohibiting eating or drinking even when labor progress is normal**	**76 (17.2)**	11 (2.5)	7 (1.6)	20 (4.6)	37 (8.5)	363 (82.8)
**Ignoring women yelling for help**	**68 (15.6)**	9 (2.1)	7 (1.6)	18 (4.0)	35 (7.9)	371 (84.4)
**Fail to arrive in time to conduct delivery**	**50 (11.4)**	5 (1.1)	2 (0.5)	7 (1.5)	36 (8.2)	389 (88.6)
**Not checking fetal heart rate until neonate is born**	**45 (10.3)**	12 (2.7)	10 (2.3)	4 (0.9)	19 (4.4)	394 (89.7)
**Using fragment of broken glass ampule or needles for AROM**^a^	**35 (7.9)**	5 (1.1)	2 (0.4)	12 (2.8)	16 (3.6)	404 (92.1)
**Charting false results to complete partograph**	**25 (5.7)**	1 (0.2)	1 (0.2)	5 (1.1)	18 (4.1)	414 (94.3)
**Pushing abdomen to rush delivery even when not an emergency**	**25 (5.7)**	1 (0.2)	1 (0.2)	3 (0.7)	20 (4.6)	414 (94.3)
**Refusing to take care of HIV-positive women**	**14 (3.2)**	7 (1.6)	2 (0.5)	1 (0.2)	4 (0.9)	425 (96.8)
**Asking for bribes for their own services**	**5 (1.1)**	1 (0.2)	0 (0.0)	0 (0.0)	4 (0.9)	434 (98.9)

^a^*AROM* Artificial Rupture of Membranes

Nine forms of D&A were reported in less than 20% of the participants. Also, four behaviors were reported by a single-digit percentage of the participants (Table 3).

**Table 3:** Multiple regression analysis of variables related to D&A behaviors

Independent variables	Dependent variable	
	D & A attitude questionnaire total	
	***β***	(95% CI)
Working hours per week	0.067	(- 0.019 ― 0.154)
Taking a break during evening shifts	0.185**	( 0.096 ― 0.273)
Professional status	- 0.171**	(- 0.266 ― - 0.076)
Interaction between nurses	- 0.153*	(- 0.248 ― - 0.058)
Any type of supervision for new nurse-midwives	- 0.091*	(- 0.177 ― - 0.006)
R^2^	0.132	

*β* standardized regression coefficients, *CI* confidence interval, *D&A* disrespect and abuse

** *P* < 0.01, * *P* < 0.05

According to the frequency distribution, participants had a higher tendency to answer “sometimes” or “several” rather than “always” or “often”, except for two behaviors: *“not draping women’s legs when performing vaginal examination”* and *“asking women about their private information in public”*.

### Factors related to D&A

To identify factors that may be related to D&A behaviors, bivariate analysis was conducted to determine the relationships of D&A scores with individual and facility factors.

There was no significant correlation between sociodemographic characteristics of the participants and D&A scores. Educational background, employment status and individual working experiences were not related to D&A scores.

Regarding working conditions and working environment, there were no factors that showed a significant correlation with D&A scores. Commute to work, having night shifts and overwork, number of days off, salary, side jobs, and frequency of breaks during each shift showed no relation with D&A behaviors. As for facility factors, facility levels, number of staff, beds, births, and deaths, assignment systems and supervision systems for educating new nurse-midwives also showed no relationship with D&A scores.

Work satisfaction of the participants was measured using Stamp’s IWS. Regarding working satisfaction, two factors of the IWS (i.e., ‘professional status’ and ‘interaction between nurses’) showed a weak correlation with D&A behaviors. There was a significant negative correlation between D&A scores and professional status (*r* = − 0.233 *p* < 0.001) and interaction between nurses (*r* = − 0.248, *p* < 0.001).

After repeating the multiple regression analysis and comparing many models that were extracted as candidates for predicting D&A behaviors, the following model was retained as the best model, which was both statistically and clinically significant and had comprehensibility (Table 3). There were no other suitable models that had notable high determination coefficients even when considering other models in comparison and these other models were also in a range of *R*^2^ < 2.0.

The results of the multiple regression analysis using total scores of D&A as a dependent variable indicated five factors related to D&A, namely, ‘working hours per week’, ‘taking a break during evening shifts’, ‘professional status’, ‘interaction between nurses’, and ‘any type of supervision for new nurse-midwives’, explaining the variance with an *R*^2^ of 0.132 (Table 3). D&A behaviors scores increased with an increase in working hours per week (*β* = 0.067) and frequency of breaks during evening shifts (*β* = 0.185). On the other hand, D&A behaviors scores decreased with an increase in scores of ‘professional status’ (*β* = − 0.171) and ‘interaction between nurses’ (*β* = − 0.153). Also, nurses and midwives who worked in facilities where there was no supervision system for new nurses and midwives scored lower than those who had supervision (*β* = − 0.091).

## Discussion

The present study is one of the few studies that focus on the self-reported D&A behaviors of healthcare providers in multiple settings of four different levels of health facilities in three different regions in Tanzania. Nearly all participants reported enacting at least one or two forms of D&A.

The most commonly reported D&A was a form of non-confidential care which involved *‘not draping women’s legs when performing a vaginal examination’*. This was the only form of D&A whose percentage of enacted behavior exceeded that of non-enacted behavior. The third commonly reported D&A was also a form of non-confidential care. According to a previous Tanzanian direct observation study, 23% (*n* = 46/197) of mothers during examination and 58% (*n* = 115/197) during birth were not provided any leg covering [19]. Another direct observation study also reported that more than half of women were not covered before birth [36]. As a number of participants in the present study worked in facilities where there was no partition between beds, privacy is a continuing concern. Sando et al. reported that almost all postpartum women (90%) shared beds in their study in Tanzania [19]. In realistic terms, facility environments in Tanzania make it more difficult to protect women’s privacy.

Non-consented care was another form of D&A that ranked high in the participant’s self-report. This is in agreement with Sando et al. who revealed by direct observation of client-provider interactions that midwives failed to get informed consent from about 80% of women before performing the procedures [19].

Nearly 30% of the participants scolded and threatened the women when they did not comply with their requirements (i.e., non-dignified care). In previous studies involving women’s reports, verbal abuse was identified as one of the most common D&A behaviors [14, 19]. Nurses and midwives commonly justified their verbal abuse believing that harsh or violent comments were necessary and unavoidable to make women obey and to ensure a safe birth [16, 37–39]. As verbal abuse was restricted to noncompliant women in these previous studies, the participants in the present study may not have also recognized verbal abuse as D&A, and scolding and threatening of noncompliant women might have become common practice.

### Risk of harm and life-threatening aspects of D&A

Although the enacted rate of D&A varied only by about 10 to 30% in the present study, participants in previous studies were conscious of engaging in physical abuse and abandonment of care, which can directly cause poor outcomes for both women and their babies. The present study showed that 35.4% of the participants performed episiotomy or suturing of perineal tears without anesthesia, whereas a previous study showed 5% [19]. Moreover, the present study showed that 5.7% of the participants pushed women’s abdomen to hasten birth even in non-emergency cases, whereas a previous study showed 3.8% [17]. Physical force was occasionally used as an aspect of corporal punishment when women did not comply with the provider’s instructions [37]. Similarly to the justification of verbal abuse, physical abuse might also have been conducted routinely.

Moreover, 10 to 15% reported abandoning their patients and exposing women and babies to the risk of death. Ignoring and neglecting women are also some of the most common D&A behaviors in Tanzania. According to women’s reports in previous studies, about 8% were ignored and neglected when they needed providers during childbirth. Many nurses and midwives reportedly showed no concern for laboring women and occasionally childbirth occurred without healthcare providers with babies dying [15, 33]. In a meta-analysis, midwives in sub-Saharan Africa took action only when women reached the second stage of labor, as they are only focused on childbirth but not on supporting women during the first stage of labor [40]. Thus, abandonment of care limits assessment of labor progress, which may lead to poor birth outcomes.

### Working conditions and systems as factors related to D&A

Our study showed that *heavy workload*, *poor relation with co-workers*, *pride of their own job* and *lack of supervision* were related to D&A behaviors of nurses and midwives, as similarly found in previous studies [16, 37, 41, 42]. However, the characteristics of the nurses and midwives and facility structural factors, which were expected to be factors related to D&A, were not significantly correlated in this study.

The factor that had the highest impact on D&A was ‘breaks during evening shifts’, which increase D&A scores proportionally. As heavy workload has been suspected to be one of the factors related to D&A behaviors of healthcare providers, taking sufficient breaks is crucial. However, the present results showed that the participants who took more breaks during evening shifts tended to enact D&A more than those who did not. As taking breaks means that nurses and midwives leave their duty and women temporarily, this variable might have been particularly related to the abandonment of care. Unbelievably, women have been reported to be left alone during childbirth while nurses and midwives take a lunch, sleep and chat [15, 43]. Inevitably, nurses and midwives who take frequent breaks will have less interaction with women, and may more frequently commit D&A such as abandonment of care.

High ‘professional status’, being proud as nurses and midwives, and having a mindset that their jobs are important and valued translate into more respectful care of women. In previous studies, professional identity and respect of nurses and midwives have been discussed as factors related to D&A, although it was not specified whether they were positively or negatively related [13, 38]. This may be associated with higher motivation of nurses and midwives for their work if they believe that their jobs are worthwhile. Thus, working without being respected and valued by others and without gratitude for the care provided could result in increased demoralization at work and the venting of frustration on women [42]. Monetary rewards may not be sufficient to afford providers with self-respect. Although financial incentives are important for motivating nurses and midwives, previous studies importantly stated that they also need to feel valued, supported and respected for their own professional value to be empowered and gain a higher self-esteem [41, 44].

Another reported factor commonly related to D&A behaviors was poor relationship among colleagues [10, 20, 45]. *‘Interaction between nurses’* showed a negative correlation with D&A. Participants who have harmonious relationships with other nurses had a lower engagement in D&A. On the other hand, a cycle of abuse may be conventional in that nurses and midwives may not attempt to treat women respectfully unless they are respected by their co-workers.

Lack of supportive supervision is also one of the most common factors related to D&A, as commonly stated in previous studies [10, 13, 20] and supported by our study. Continuing education regardless of type for starting nurses and midwives may ameliorate D&A behaviors. Supervision systems can make new nurses and midwives feel supported by senior staff who can become role models. All the more, interactions rather than supervision systems are anticipated to bring affective changes that can positively influence behaviors of nurses and midwives to women. Although the impact of ‘working hours’ on D&A behaviors was the smallest, long working hours have also been argued in other studies as a factor related to more D&A [6, 37]. Working long hours may cause not only fatigue among nurses and midwives, but also decreased motivation and job satisfaction [41], leading to uncaring behaviors towards women [10].

### Implementation of changes at the individual and working environment levels

Based on our analysis, evidence indicates that satisfying working conditions and optimal supervision systems including respect for healthcare providers and harmonious personal relationships are important in reducing D&A. It may be difficult to reduce D&A behaviors of healthcare providers by changing their morals through their own individual efforts alone. However, fostering a pleasant working atmosphere, good working conditions and systems, and a professional and respectful facility culture exemplified by harmonious relationships among colleagues, patients and the community is crucial in assisting healthcare providers function optimally with a sense of respect and kindness. Working in an atmosphere wherein healthcare providers feel respected by others will affect how they treat women respectfully. Understanding both drivers and context of D&A is needed rather than blaming the attitudes and behaviors of individual healthcare workers [40]. As diverse factors are implicated in the development and complex context of D&A, avoidance of blaming only healthcare providers is paramount. In the context of D&A occurrence, both the provider’s individual and facility environment factors were conducive to develop disrespectful behaviors [19, 20].

### Limitations and strengths

This study has some limitations. Retrospective studies can have a recall bias wherein the participants may have a faulty recall of their D&A events as a potential limitation. The questionnaire for measuring D&A behaviors had only one to three items to identify each category and overall internal consistency was moderate. The next step to consider is scale refinement. The psychometric properties of the scale should be considered for further development such as factor analysis. As this was an initial study, a more encompassing or complex model for predicting D&A behaviors was beyond the scope of this work. It was also possible to explain D&A behaviors using other factors that are assumed to be related to D&A but not included in this study. These include provider’s perception of women, provider’s own history of being abused, and the culture of the facility.

Regarding the strengths of the present study, this is one of the few studies of self-reported D&A behaviors of healthcare providers. Moreover, the data collection covered four levels of public health facilities in three regions of Tanzania. Thus, involvement of multiple facilities and regions in Tanzania indicates its strength in terms of external validity.

Notably, D&A is an important issue faced not only by Tanzania and other low-income countries, but also by high-income countries. As healthcare providers involved with childbirth care are not only nurses and midwives, further research on D&A targeting obstetricians and other providers is warranted.

## Conclusion

Nearly all nurses and midwives who participated in this study in Tanzania reported enacting at least one or two forms of D&A during childbirth. The most prevalent D&A behaviors engaged in by a relatively large proportion of nurses and midwives were non-confidential care, non-consented care and non-dignified care including verbal abuse. These D&A behaviors might have a psychological effect on women’s childbirth experience. Less prevalent D&A behaviors engaged in by nurses and midwives were physical abuse and abandonment of care. These have potential life-threatening impact on women and newborns.

Five factors were found to be related to D&A behaviors of nurses and midwives as indicated by D&A scores. D&A scores increased with an increase in ‘**working hours per week**’ and ‘**taking a break during evening shifts**’. D&A scores decreased with an increase in the scores of the ‘two components of IWS (**professional status** and **interaction between nurses**)’, and ‘**any type of supervision for new nurse-midwives**’. Almost all other demographic characteristics of nurses and midwives were not significantly correlated with D&A. Working conditions and the healthcare system were related to D&A behaviors of nurses and midwives rather than their individual and facility structural characteristics.

## Supplementary information

**Additional file 1.**

## Data Availability

All data generated or analyzed during this study are included in this published article.

## Abbreviations

D&A: Disrespect and Abuse
SDGs: Sustainable Development Goals
RAs: Research Assistants
IWS: Index of Working Satisfaction
